# Supplementary material for: Assessing the Policy gaps for achieving China’s climate targets in the Paris Agreement
Source: Nat Commun. 2019 Mar 26;10:1256. doi: 10.1038/s41467-019-09159-0 (PMC6435737; doi:10.1038/s41467-019-09159-0)
Supplement: Supplementary file 1 — Supplementary Information [file 41467_2019_9159_MOESM1_ESM.pdf]

Supplementary Notes

Supplementary Note 1: The survey protocol

Article: Assessing the Policy Gaps for Achieving China's Climate Targets in the Paris Agreement.

Authors: Gallagher et al.

This survey protocol is also available through [https://figshare.com/s/483d43b02d6110c8ea24]

Expert Elicitation for Climate Policy Impact Analysis for China

Kelly Sims Gallagher and Fang Zhang

April 7, 2017

Dear expert:

Thank you for agreeing to participate in our expert elicitation about the impacts of climate policy in China. The aim of the study is to determine the estimated impact of China's existing and planned policies, as well as additional policies that might needed to meet or beat China's targets under the 2015 Paris Agreement on Climate Change (see Appendix A). Your expertise is highly valued and we greatly appreciate your time.

You are requested to attempt to answer these questions in writing and return them to us by email by May 15th. After receiving your survey response (by email to kelly.gallagher@tufts.edu or fang.zhang@tufts.edu), Prof. Kelly Sims Gallagher and/or Dr. Fang Zhang may follow up to clarify your answers. Professor Gallagher leads the Climate Policy Lab at the Center for International Environment and Resource Policy at The Fletcher School, Tufts University. Dr. Zhang is a research fellow in the Climate Policy Lab.

This expert elicitation is one of multiple methodologies will be used to conduct the analysis. We will conduct an independent analysis of all the data collected using different methods, and then publish a discussion paper. We will send the discussion paper to you for feedback, and ultimatelyplan to submit a final paper to a peer-reviewed journal. Your name will be listed at the end of the report as one of the experts consulted, but your name will not be identified specifically with any single answer. If you would prefer to be listed anonymously, please check off the box in question 2.

Thank you again for taking the time to participate in this expert elicitation.

Kelly Sims Gallagher and Fang Zhang

Questions

1. Please provide your name, title, and affiliation.

2. Would you prefer to be listed anonymously as a expert?
   a. Yes ☐
   b. No☐

3. Using a scale of 1-10 where 1 is "easy" and 10 is "almost impossible", please estimate how difficult you believe it will be for China to achieve the targets set by China in its Nationally-Determined Contribution (NDC) by 2030 (please see Appendix A for the list of targets). Please explain why you believe each target will be easy or difficult.

   a. Peaking carbon dioxide emissions around 2030 _____

   b. Peaking early (well advance of 2030) _____

   c. Lowering carbon dioxide emissions per unit of GDP by 60% to 65% from the 2005 level _____

   d. Increasing the share of non-fossil fuels in primary energy consumption to around 20% _____

   e. Increasing the forest stock volume by around 4.5 billion cubic meters on the 2005 level _____

4. Based on your expertise, what year do you predict that China's carbon dioxide emissions will peak will occur (your best guess is fine)? Why?

5. Do you predict that China's emissions will plateau (peak but not decline)? If so, for how many years do you expect there to be a plateau? Why?

6. In Appendix B, a simplified list of China's major national policies on climate change has been provided. Please choose from this list the top five policies that you believe have been most impactful in already reducing carbon dioxide emissions in China, and please rank them in terms of most effective to least effective. If a policy you believe has been highly effective (worthy of being in the top 5) that is not on the list, please write it in.

    a. Most effective policy:

    b. 2nd-most effective policy:

    c. 3rd-most effective policy:

    d. 4th-most effective policy:

    e. 5th-most effective policy

7. Are there any policies not included in the appendix that you believe to have substantially reduced carbon dioxide emissions, even if you would not rank them to be in the top 5? If so, please list them here, along with the years they were implemented and in force.

8. For each of the policies you listed in your answer to question 4, can you provide a quantitative estimate of the carbon dioxide emissions reductions achieved per year? Recognizing this is a difficult question, please answer it however you can (e.g. as a percentage from a base year, as a reduction from business-as-usual, as an absolute reduction in millions of tons of CO_2/year)? Please specify your timeframe. If there is a source for your data, please provide it accordingly. The goal of this question is to begin to quantitatively estimate the impacts of different policies.

	Estimated quantitative impact of policy		
		**Units** (e.g. million tons CO_2, percentage reduction, kWh, GW capacity, or other)	**Year(s)**

**Most effective policy**			
**2nd most effective policy**			
**3rd most effective policy**			
**4th most effective policy**			
**5th most effective policy**			

9. Of the top five policies you have identified above, do you believe there is potential overlap or redundancy among them, and if so, how much?

10. Of any of the policies you have identified above, do you believe there will be a significant discrepancy between the reductions achieved in theory versus in practice? For each policy, please provide the percentage of policy impact lost through implementation. Your answer does not need to be quantitatively precise, so please use the options provided below:

	Percentage of theoretical emission reduction impact lost during practical implementation process				
	**0-20% loss**	**20-40% loss**	**40-60% loss**	**60-80% loss**	**80-100% loss**
**Most effective policy**					
**2nd most effective policy**					
**3rd most effective policy**					
**4th most effective policy**					

**5th most effective policy**					

11. Do you believe that the policy gap between the climate policies that currently exist and those that would be required to achieve China's goal to peak emissions by 2030 is:

    a. Very large, almost impossible to overcome with new and additional policies
    b. Large, can overcome with serious and deliberate effort
    c. Medium, achievable with modest effort to fix existing or implement new policies
    d. Small, very easy to achieve with existing policies

12. Which existing policies do you believe could be improved to achieve greater emissions reductions through revision or different means of implementation? Why? For each policy you recommend revising, what specific changes would you recommend that the Chinese government make to these policies to improve the likelihood that China will achieve its climate targets?

13. Which new and additional policies would you recommend to the Chinese government in order for China to achieve its climate targets, and why? Please rank them in order of likely effectiveness?

14. Which national Chinese climate policies would you recommend be eliminated because they are either ineffective from a mitigation point of view or too expensive in terms of cost-benefit analysis.

15. The Chinese NDC targets are primarily focused on mitigation of carbon dioxide emissions. Do you see potential for mitigation for other greenhouse gases? If so, which specific policies would you recommend be considered by the Chinese government?

16. Do you have any specific suggestions for how the Chinese government could achieve synergies between mitigation and adaptation/resilience policies?

17. Do you observe any tensions between mitigation and adaptation policies in China?

Appendix A

In 2014, China first announced its intended nationally determined contribution (INDC) for the Paris Agreement on Climate Change in the context of a joint announcement between President Barack Obama and President Xi Jinping. It later formalized its commitment through its submission to the UN Framework Convention on Climate Change.[1] The key commitments are excerpted from the submission below:

> "Based on its national circumstances, development stage, sustainable development strategy and international responsibility, China has nationally determined its actions by 2030 as follows:

[1] The formal submission is available at this link: http://www4.unfccc.int/Submissions/INDC/Published%20Documents/China/1/China's%20INDC%20-%20on%2030%20June%202015.pdf

- To achieve the peaking of carbon dioxide emissions around 2030 and making best efforts to peak early;
- To lower carbon dioxide emissions per unit of GDP by 60% to 65% from the 2005 level;
- To increase the share of non-fossil fuels in primary energy consumption to around 20%; and
- To increase the forest stock volume by around 4.5 billion cubic meters on the 2005 level.

Moreover, China will continue to proactively adapt to climate change by enhancing mechanisms and capacities to effectively defend against climate change risks in key areas such as agriculture, forestry and water resources, as well as in cities, coastal and ecologically vulnerable areas and to progressively strengthen early warning and emergency response systems and disaster prevention and reduction mechanisms."

Appendix A: Major National Climate Policies in China	
Key policies	*Details*
**Economy wide**	
1. S&T on Climate Change	*Increase R&D and science capacity in climate change fields.*
2. Energy-intensity target	*15% below 2015 levels by 2020*
3. Carbon-intensity target	*18% below 2015 levels by 2020；60-65% below 2005 by 2030*
4. National Emission Trading Sysfem	*Upgrading the regional ETS system to a national ETS*
**Transportation**	
5. Vehicle efficiency standards	*Light duty vehicle efficiency standards (47 mpg or 5 L/100 km) (MIIT)*<br>*Heavy duty vehicle efficiency standards (MIIT)*<br>*Commercial vehicle efficiency standards (MIIT)*<br>*Encouraging small-displacement energy saving cars*
6. Guiding Opinions on Establishing s Low-Carbon Transport System	- *1) Operating vehicles: Energy consumption per unit of transport volume should fall 10% by 2015 and 16% by 2020 from 2005 levels; CO2 emissions per unit of transport volume should fall 11% by 2015 and 18% by 2020 from 2005 levels. 2) Operating ships: Energy consumption per unit of transport volume should fall 15% by 2015 and 20% by 2020 from 2005 levels; CO2 emissions per unit of transport volume should fall 16% by 2015 and 22% by 2020. 3) Urban transport per person: Energy consumption should fall 18% by 2015 and 26% by 2020 from 2005 levels; CO2 emissions intensity should fall by 20% by 2015 and 30% by 2020 from 2005 levels.*
7. Promoting the Use of New Energy Vehicles with purchase subsidies	*1) EV cars: 25,000-55,000RMB; 2)Passenger car: 120,000-500,000RMB; 3) EV or Hyd carriage trucks: 1800/KWH; 4) fuel EV: 200,000-500,000 per car; 5) reduce subsidies by 20% in 2017-2018 based on that in 2016; 2019-2020, reduced by 40% based on 2016.*
Power	

8. Phasing out small and outdated coal-power capacity	*Phase out small (<50 MW), inefficient coal plants;*<br>*13th FYP for Electricity Sector Pledges to remove 20GW outdated coal-power capacity*
9. Coal consumption cap	*Coal consumption cap of 4.1 Gt by 2020 and the share of coal in primary energy consumption should be no more than 58% by 2020*
10. Energy Efficiency Standards for Coal Power(2014-2020)	*Average coal consumption of new coal-fired power plants should not be more than 300g of standard coal per kWh and the average coal consumption of existing upgraded coal-fired power plants should not be more than 310g of standard coal per kWh[Average standard coal equivalent consumption per kWh for coal power generation was 323 g/kWh in 2013]*
11. Resource tax	*1) crude oil: 5-10%; 2) natural gas: 5-10%; coal-choking coal 8-20/ton, other coal 0.3-5 per ton, etc.*
12. Natural Gas Policy	*- Natural gas consumption is 8.3~10% of the primary energy consumption by 2020 (2015:5.9%)*<br>*- Subsidy for exploring and utilizing shale gas: 0.3 RMB/M3 from 2016-2018; 0.2 RMB/M3 from 2019-2020.*<br>*- Shale gas industry defined as a new "strategic" industry, setting general policies for shale gas development.*
13. Non-fossil energy consumption as 15% of total energy consumption by 2020	*-Non-fossil energy targets of 210-250 GW for wind, 110-150 GW for solar, 340 GW for hydro, 58 GW for nuclear*<br>*- Mid to Long-Term Development Plan for Nuclear Energy (2011-2020): nuclear operating capacity amount to 40GW by 2020; nuclear electricity reaches to 260-280 billion kWh per year. The nuclear capacity under construction should be 18GW by the end of 2020.*
14. Feed-in-tariffs for renewable energy development	*Wind ranging as of 1 Jan 2017 from RMB 0.4/kWh to 0.57/kWh depending on category for onshore wind;*<br>*Offshore wind: RMB 0.85 to 0.75/kWh*<br>*Solar: starting from 1 Jan 2017, RMB 0.65, 0.75 and 0.85 per kWh according to the solar resources zone.*<br>*Distributed solar PV remain unchanged and rests at RMB 0.42 per kWh.*<br>*Feed-in tariff for all renewables (2012)*<br>*- Surcharging 0.008 RMB/KWH as RE development fund to support clean energy.*
15. Green Energy Certificate	*Power generators should have 9% of their electricity generation coming from non-hydro renewable energy. To achieve this goal, encourage using renewable energy certificate to trade in ETS system or Energy Saving systems,*

16. Tax incentives for renewable energy	*Half Value-added tax for wind and solar power sector. That's charging only 7.5% VAT.*<br>*Exempting income tax for first three years and half for the second three three years for enterprises engaging in projects involving power stations that use renewable energy, including solar power (2008)*
17. Electricity regime reform	*Several Guidance on further Deepening Electricity Regime Reform (NO. 9 Document): fully open DG power market, e.g. solar power, wind power, biomass, etc.; increasing the grid connection for new and renewable energy, energy efficiency projects, etc.*
18. Removing Fossil Fuel Subsidies	*- China is planning to issue clean time schedules as promised in G20 2016*
**Industrial**	
19. Industrial Transformation and Upgrading Plan	*Accelerating the structural adjustment of industries and eliminating outdated and energy-intensive industries (i.e. cement, aluminum, chemicals, coking, petrochemicals, glass, steel*<br>*- State Council Instruction Opinion on Solving the Problem in Overcapacity*<br>*- Phasing out small and outdated industrial production capacity*<br>*- Phase out small coal mines, coking plants, and iron producers. Expanded to 19 industries in 2011.*<br>*- 13FYP, Phasing out outdated production capacity of coal 0.8 billion tons per year and add advancing production capacity by 0.5 billion tons by replacing. Phasing out outdated production capacity of crude steel by 100-150 million tons by 2020*<br>*- Providing central fiscal subsidies for phaseout out outdated production capacity*
20. Differentiated electricity tariffs	*Differentiated electricity pricing policy for high energy intensive sectors: Encouraged and permitted enterprises pay the normal price for electricity while enterprises in the restricted and eliminated categories pay surcharges of 5 fen and 20 fen per kWh ($0.007/kWh and $0.029/kWh), respectively, which is about 10% and 30% of the average price of electricity per kWh. In 201, 2014 and 2017, further increasing electricity prices for cement and iron and steel firms.*
[illegible]	*Energy Efficiency Standards for electronic motors: GB18613-2012; GB30254-2013.*<br>*Energy Efficiency for Industrial Boiler, GB24500-2009*
	Guidance on Strengthening Energy Conservation of Internal-combustion Engine: energy saving internal combustion engine takes up to 60% of the whole owned engined in China

21. Energy efficiency standards and labeling for industrial equipment	*Issued four lists of Product Catalogue of Outdated Electronic motors*
	Catalogues of Recommended Energy-Saving Electromechanical Equipment (Products) and energy star
	Catalogue of Obsolete Mechanical and Electrical Equipment to be Eliminated due to High Energy Consumption
	Action Plan for Industrialization of High Energy Saving and Environment Protection of Industrial Boiler
22. Key Enterprises Program	*Top -1000 Enterprise Program in 11th FYP, Expanded to top-10,000 enterprises in 2011 and Top 100-1000-10,000 enterprises action in 13th FYP*
23. Energy Saving and Emission Reduction Subsidy Funding	*Specific funding for specific usage; key supporting areas: institutional innovation; basic capacity and public platform building; key area, key sectors and key region, etc [this policy replacing energy saving subsidy policies, e.g. 300RMB/ton subsidies for energy conservation]. There are multiple subsidy formats: awards, subsidy, interest subsidy, etc.*
**Green Finance**	
24. Green Credit Policy	*- No new credit for firms which failed to pass environment impact evaluation, and firms from limited or outdated sectors, etc.; EPA provides environment data to banks.*<br>*- limited credits to high pollution sectors, and also encouraging credits to clean sectors and energy efficiency projects*
25. Green Bond Policy	*- Green Bond Issuing Guideline*<br>*- Supporting Project List of Green Bond: clarifying 6 major sectors and 31 sub-sectors*<br>*- Allowing firms use no more than 50% of bonds to pay bank loans or operation finance.*
26. Guidelines for establishing Green Finance System	*- Promoting green credit, using securities to support green investment, establishing green development fund, developing green insurance, amplifying environment right exchange market and finance tools, advancing international cooperation, etc.*
**Land use**	
27. Forest Building Planning	*increase forest volume to 16.5 billion meters^3, and the forest coverage rate up to 23.4% by 2020*<br>*increase forest volume to 23 billion meters^3 by 2050, the forest coverage rate up to 26% by 2050.*

28. Loan preferential policy for forest project	*Develop banks and agriculture development bank should provide long-term loans to forest project, which can be up to 30 years. The loan interest rates should be low.*
29. Financial subsidies for forest	*1) subsidies for forest ecological effect, 10-20/Mou; 2) subsidies for young forest spring cultivation*
30. Encouraging farmers giving up farming to rebuild forests and grass ground (退耕还林):	*- 1500/ mou for forest recoverting; 1000/mou for grass retreating.*
**Residential and Commercial**	
31. Design energy efficiency standard (technical standards for floors, directions, wall and window insulations, heating and cooling systems, etc)	- *Residential buildings in hot summer and cold winter zone. The goal is to reduce 50% energy consumption for new buildings relative to old versions*<br>- *Residential buildings in severe cold and cold zones. The goal is to reduce 65% energy consumption for new buildings relative to old versions*<br>- *Residential buildings in hot summer and warm winter zone. The goal is to reduce 65% energy consumption for new buildings relative to old versions*<br>- *Public Buildings in different climate zone. The man focus is on air condition system. The goal is to reduce 50% energy consumption for new buildings relative to old versions*
32. Green Building Policy	- *Increasing the ratio of green buildings in new added buildings over 30% by 2020;*<br>- *Setting assessment standard for green building. GB/T 50378-2014*<br>- *Label green buildings into three types based on the total score: one star, two star and three star*<br>- *Providing subsidies for green building: 2 star green building- 45Yuan/M2, 3 star 80Yuan/M2 in 2012; scaling up green buildings and establishing green eco-urban: 50 million/ green eco-urban*<br>- *Setting up green building innovation award:*

33. Energy Information Labeling Program for Appliance	- *Measures for the Administration of Energy Efficiency Labels*<br>- *The Product Lists for China Energy Label: really published for twelve lists, including: refrigerators, TVs, Air Conditioners, Wind Fans, converters, lights, computers, printers, fax machines, etc.*<br>- *Energy Efficiency Star Certified Products List (voluntary in nature, promotes high energy-efficient products)*<br>- *Energy Efficiency "Pioneer" Label. The first list is in 2016*<br>- *Procedures for Low-Carbon Product Certification Management. Has already published two lists.*
34.Building lighting	*Standard for lighting design of buildings- GB50034*
	China's Road Map of Phasing Out Incandescent Lamp: since 2016, forbidding importing and selling >=15 W Incandescent Lamp in China. According to NDRC, energy efficient lamp will save 60%-80% electricity relative to incandescent lamp.
35. Government procurement	*Mandatory requirements for government to procure energy efficient products. NDRC and MoF*<br>*Lists of Energy Efficient Products for Government Procurements. Already published twenty times until 2016*
