# Supplementary material for: Assessing the Policy gaps for achieving China’s climate targets in the Paris Agreement
Source: Nat Commun. 2019 Mar 26;10:1256. doi: 10.1038/s41467-019-09159-0 (PMC6435737; doi:10.1038/s41467-019-09159-0)
Supplement: Supplementary file 3 — Description of Additional Supplementary Files [file 41467_2019_9159_MOESM3_ESM.pdf]

Description of Additional Supplementary Information

File Name: Supplementary Data 1

Description: A comprehensive policy inventory of China's current and forthcoming climate change policies that we developed. We assembled the inventory mainly from primary sources, namely government documents issued by each relevant government ministry in China. Secondary sources, such as the International Energy Agency's Policies & Measures database, were also used. The policy inventory yielded more than 100 separate climate policies at the national level in China. We classified those policies by type, such as regulatory/administrative, fiscal, market-based, informative, innovation, diplomatic, and other. Some policies fit into more than one of these categories.
