# Supplementary material for: Assessing the Policy gaps for achieving China’s climate targets in the Paris Agreement
Source: Nat Commun. 2019 Mar 26;10:1256. doi: 10.1038/s41467-019-09159-0 (PMC6435737; doi:10.1038/s41467-019-09159-0)
Supplement: Supplementary file 4 — Supplementary Data 1 [file 41467_2019_9159_MOESM4_ESM.pdf]

Supplementary Data 1 The inventory of Chinese climate policies

Article: Assessing the Policy Gaps for Achieving China's Climate Targets in the Paris Agreement.

Authors: Gallagher et al

The Supplementary Data 1 is a comprehensive policy inventory of China's current and forthcoming climate change policies that we developed. We assembled the inventory mainly from primary sources, namely government documents issued by each relevant government ministry in China. Secondary sources, such as the International Energy Agency's Policies & Measures database, were also used. The policy inventory yielded more than 100 separate climate policies at the national level in China. We classified those policies by type, such as regulatory/administrative, fiscal, market-based, informative, innovation, diplomatic, and other. Some policies fit into more than one of these categories.

The data is also available through [https://figshare.com/s/3cc9d39b26155714b0eb].

Table 1. Inventory of Chinese Climate Policies

**Major National Climate Policies in China Since 2000**				
Sector	*Policy*	*Dates*	*Type*	*Source*
**Economy wide**				
	**Specially Designated National Plan on Science and Technology Development in Tackling Climate Change during the 13th FYP**<br>The Plan is jointly released by MOST, MEP and CMA.	Updated in 2017, first issued in 2012 for the 12th Five-Year Plan Period	Innovation	MOST, MEP & CMA [2017] No.120
	**The 13th Five Year Plan on Energy Development (2016-2020)**<br>The latest Plan provides an update on the targets set in the ***Energy Development Strategy Action Plan (2014-2020)***.<br><br>The new targets include a cap on annual primary energy consumption set at 5.0bn tonnes of the standard coal equivalent by 2020, with a need to limit the annual growth rate of primary energy consumption to 2.5 percent. The annual coal consumption should be held below 4.1bn tonnes until 2020.<br><br>The share of non-fossil fuels in the total primary energy mix is to rise to more than 15% by 2020. The share of natural gas should reach 10 percent, while that of coal will be reduced below 58 percent. In addition, installed nuclear power capacity is to reach 58GW by 2020, with additional 30GW expected to be under construction in 2020. Installed capacity of hydro-, wind and solar power in 2020 is expected to reach at least 340GW (plus 40 GW pumped storage power), 210GW (205GW online, 5GW offshore) and 110GW (including more than 60 GW of distributed solar energy systems and 5GW of thermal solar), respectively. Energy self-sufficiency should be above 80 percent.	2016	Regulatory Plan	No. 31 [2014] of the State Council; NDRC

	In addition, China aims to reduce carbon dioxide emissions per unit of GDP by 18 percent from 2015 levels by 2020.			
	**Energy Technology Revolutionary Innovation Action Plan (2016-2030)**<br>The objective of the Plan is that by 2020, China should see a significant improvement in independent energy innovation, with major breakthroughs in key technologies and a decrease in foreign dependence for energy technology and equipment, key components and materials. By the year 2030, a sound energy technology innovation system will be in place, with a capacity to support coordinated and sustainable development of China's energy industry. By then, China should be among the global powers in energy technology. The Action Plan also includes a ***Roadmap of Key Innovation Actions for Energy Technology Revolution***, putting forward innovative objectives for 2020, 2030 and 2050 respectively.	2016	Innovation Plan	NDRC Energy [2016] No.53
	**China 13th Energy Technology Innovation Five Year Plan (2016-2020)**	2016	Innovation Plan	NEA Technology [2016] No. 397
	**Work Plan for the Pilot Construction of Climate Resilient Cities**<br>Jointly released by the NDRC and the MOHURD, the Plan proposes to incorporate climate resilience indexes into the urban-rural planning system, construction plans and industrial development plans, build 30 climate-resilient pilot cities, improve average cities' climate-resilient management and raise the proportion of green buildings to 50% by the year 2020.	2016	Plan	NDRC Climate [2016] No.245
	**Work Plan for Greenhouse Gas Emission Control during the 13th Five-Year Plan Period (2016-2020)**<br>It aims to lower carbon dioxide emission per GDP unit by 18% of 2015 emission level by 2020	2016, first issued in 2011 for the 12th Five-Year Plan Period	Plan	No. 61 [2016] of the State Council

	**Comprehensive Work Plan on Energy Conservation and Emission Reduction**<br>Mandatory energy intensity reduction targets were first allocated to local governments in 2007. The latest Work Plan published for the 13th FYP sets forth that by 2020, the nation energy consumption per 10,000 RMB of gross domestic product (GDP) will be reduced by 15% compared with 2015; the total energy consumption will be capped at 5 billion tonnes of standard coal; and the total volatile organic compounds (VOC) emissions across the whole nation will be cut by more than 10% compared with 2015.<br>The latest Work Plan also initiates the ***"100-1,000-10,000" Energy Conservation Program***, which aims to put the top 100 energy consuming enterprises in China under regulation of the central government, the top 1,000 energy consuming enterprises under the regulation of their respective provincial-level governments, and a further 10,000 plus high-energy consuming enterprises under the regulation of lower-level governments.	2016, first published in 2007 and then updated in 2011	Regulatory	No. 74 [2016] of the State Council
	**Energy Conservation Law**	Revised in 2016, first issued in 1997	Law	State Council Presidential Order 48
	**Administrative Measures for Energy Efficiency Labels**<br>China's national energy efficiency labeling system started with refrigerators and air conditioners. The revised Measures specifies the information to be included in the energy efficiency labels and requests that manufacturers and importers use the labels on energy-consuming products listed in the corresponding catalogue.	2016, first introduced in 2004	Informative, Regulatory	NDRC & AQSIQ [2016] No.35,
	**Opinions of the CPC Central Committee and the State Council on Further Promoting the Development of Ecological Civilization**	2015	Guideline	No. 12 [2015] of the CPC Central Committee

	**Overall Plan for the Structural Reform for Ecological Civilization**<br>The Plan aims at gradually establishing the control system and the implementation mechanism of national total carbon emissions, establishing an effective mechanism to increase the forests, grasslands, wetlands, and ocean carbon sink, and strengthening international cooperation in response to climate change.	2015	Plan	No. 25 [2015] of the CPC Central Committee
	**National Plan on Climate Change (2014-2020)**<br>The Plan sets a target of reducing carbon emissions per unit of GDP by 40-45% from 2005 level by 2020, increasing the percentage of non-fossil fuels in primary energy consumption to 15% and increasing the proportion of forest area and stock volume by 40m ha and 1.3m m3 respectively from a 2005 baseline.	2014	Plan	NDRC Climate [2014] No. 2347
	**Notice on Organizing and Promoting Key Enterprises and Public Institutions to Report Greenhouse Gas Emissions**	2014	Regulatory	NDRC Climate [2014] No.63
	**National Strategy for Climate Adaptation**<br>lays out clear guidelines and principles for climate change adaptation and proposes some specific adaptation goals	2013	Plan	NDRC Climate [2013] No.2252
	**HCFC Phase-out Management Plan (HPMP)**<br>Since 2011, China has been implementing the first stage of its HPMP in industrial and commercial refrigeration, targeting at phasing out 3,386 ODP tonnes of HCFCs by 2015. The Chinese government also finalized proposal for the Stage II HPMP in 2016, with a focus on natural refrigerant technologies. Stage II proposes a phase out of 4,749 ODP tonnes of HCFCs by 2020, and an additional 4,684 ODP tonnes by 2026 to assist the Government of China in meeting the 35 per cent and the 67.5 per cent reduction targets by 2020 and 2025, respectively.<br><br>The MEP issued a ***Circular on Strict Management of HCFC Production, Sales and Consumption*** in 2013, requiring quota permits from all enterprises producing HCFCs and consuming over 100 metric tonnes (mt) of HCFCs, and registration at	2011	Regulatory	MEP [2013] No.179

	local Environmental Protection Bureaus for enterprises consuming less than 100 mt.			
	**Circular Economy Promotion Law**	2008	Law	State Council Presidential Order 4
	**National Climate Change Programme**<br>The Programme, China's first global warming policy initiative, outlines objectives, basic principles, key areas of actions, as well as policies and measures to address climate change for the period up to 2010.	2007	Guideline	No.17 [2007] of the State Council
	**Promotion of Circular Economy**<br>In 2005, the State Council issued **Suggestions on Accelerating the Development of Circular Economy**, which was China's first document that supported promotion of circular economy implementation from a national level. In 2010, the NDRC issued the ***Guidelines for Making Plans for Circular Economy Development***, suggesting that local governments should develop the circular economy according to their specific circumstances. In 2013, *the State Council issued the **Development Strategy and Immediate Action Plan of Circular Economy***, setting goals for China's circular economy development in different stages. The NDRC also issued the ***Plan for the Promotion of Circular Economy*** in 2014 and 2015, which include actions and targets to use resources (water, metals, land and coal) more efficiently and to better manage resources and waste in industries, agriculture and cities. In addition, The MIIT has released six editions of the ***Catalogue of Remanufactured Products*** to promote the use of remanufactured products.	2005	Guideline	No.22 [2005] of the State Council, NDRC [2010] No. 311, No. 5 [2013] of the State Council, NDRC Environment and Resources [2015] No.769, MIIT [2016] No.67
	**Medium and Long Term Energy Conservation Plan**<br>The Plan includes energy conservation targets up to 2020. Energy consumption per 10,000 Yuan GDP is expected to drop to 1.54tce in 2020, with an annual average energy conservation rate of 3% from 2003 to 2020. And by 2020, energy consumption per unit of major product production is expected to reach or approach the international advanced level. It also identifies key fields and key projects for improving energy efficiency.	2004	Plan	NDRC Environment and Resources [2004] No. 2505

Transportation				
	**The 13th Five-year Plan for Energy Conservation and Emissions Reduction in Road and Water Transport**<br>The Plan provides specific energy consumption and CO_2 emissions reduction goals for different modes of transport for 2020:<br><br>For operating vehicles - Energy consumption per unit of transport volume should fall 10% by 2015 and 16% by 2020 from 2005 levels; CO2 emissions per unit of transport volume should fall 11% by 2015 and 18% by 2020 from 2005 levels.<br><br>For operating ships: Energy consumption per unit of transport volume should fall 15% by 2015 and 20% by 2020 from 2005 levels; CO2 emissions per unit of transport volume should fall 16% by 2015 and 22% by 2020.<br><br>For urban transport per person: Energy consumption should fall 18% by 2015 and 26% by 2020 from 2005 levels; CO2 emissions intensity should fall by 20% by 2015 and 30% by 2020 from 2005 levels.<br><br>Prior to this, China also issued the ***Medium and Long-Term Outline about Energy Conservation in the Fields of Road and Water Transportation*** in 2008, ***Guiding Opinions on Establishing a Low-Carbon Transport System*** and the ***12th Five-year Plan for Energy Conservation and Emissions Reduction in Road and Water Transportation*** in 2011.	2016	Plan	MOT [2016] No. 94
	**R&D Program of New-Energy Vehicles (2016-2020)**<br>Electric Vehicle Major Project has been an important component of the 863 Program since the 10th five-year plan period.<br><br>In 2015, the MOST solicited opinions for a plan to support the research and development of new energy vehicles as a key major project over the next five years. According to the plan,	2016, first introduced in 2001	Innovation	MOST [2016] No.305

	in 2020, the battery module should have an energy intensity of 300 Wh/kg or more and FCEV is to achieve "thousands" market scale in 2020.			
	**Adjustment to Subsidies for New Energy Vehicles**<br>The Policy Adjustment was jointly issued by MOF, MOST, MIIT and NDRC in December 2016. It supplements the ***Financial Support Policy for New-energy Vehicles, 2016-2020*** issued in 2015 and represents the sixth adjustment to the original policy introduced in 2009. It requests that subsidies for pure electric vehicles and plug-in hybrid vehicles to be reduced by 20 percent in 2017 to 2018 from that in 2016, and 40 percent in 2019 to 2020 from that in 2016.<br><br>Following this adjustment, the subsidies from central government range from 20,000-44,000 yuan per vehicle for pure-electric and hybrid electric vehicles. Subsidy standards for fuel cell electric vehicles remain unchanged, ranging from 200,000 to 500,000 yuan per car.<br>In September 2013, the MOF announced a long-anticipated renewal of China's electric vehicle subsidies. Consumers who purchases EVs can get up to 60,000 yuan ($9,400) for pure electric cars with a range over 250 km, and 50,000 yuan and 35,000 yuan for EVs with range over 150 km and 80 km, respectively.	2016, first introduced in 2009 and then updated in 2013 and 2015	Fiscal	MOF [2016] No. 958
	**Emission Standards for New Passenger Cars and Light-duty Commercial Vehicles**<br>In December 2016, MEP released the final rule of the Stage 6 Limits and Measurement Methods for Emissions from Light-Duty Vehicles. The China 6 standard, which is to take effect beginning on July 1, 2020, is one of the most stringent emission standards around the world for the post-2020 time frame. Unlike the previous standard phases, which closely follow the European emission standards, the China 6 standard combines best practices from both European and U.S. regulatory requirements (California regulations) in addition to creating its own.	2016, first introduced in 1999	Regulatory	MEP [2016] No. 79

	**Tax-exemption Policy for New-Energy Vehicles**<br>In 2015, a new tax-exemption policy was jointly issued by MOF, MIIT and the State Administration of Taxation. It is an updated version of the policy introduced in 2012. According to this policy, new-energy vehicles and ships will be exempted from vehicle and vessel tax.	2015, first introduced in 2012	Fiscal	MOF, SAT & MIIT [2015] No.51
	**Demonstration and Promotion of Energy Efficient and Alternative Energy Vehicles**<br>It was first known as the "Ten Cities, Thousand Vehicles Program", which was to stimulate electric vehicle development through large-scale pilots in ten cities with government subsidies, focusing on deployment of electric vehicles for public fleet applications. The Program has since been expanded to 88 cities.<br><br>The ***Opinions on Accelerating the Promotion of the Application of New Energy Vehicles in the Transportation Industry*** released by the MOT in 2015 set a target of 300,000 new energy vehicles on China's roads by 2020: 200,000 new energy buses and 100,000 new energy taxis and delivery vehicles.	2015, first introduced in 2009 and then updated in 2013 and 2014.	Fiscal	MOF & MOST [2009] No.6, MOT [2015] No.34
	**Vehicle Fuel Economy Standards**<br>China's first-ever fuel consumption standards for passenger vehicles were adopted in 2004. The latest Phase 4 Passenger Car Fuel Consumption Standard released by the MIIT in 2014 regulates domestically manufactured and imported new passenger cars sold in China from 2016 to 2020. It projects an overall fleet-average fuel consumption of 5L/100km for new passenger cars in 2020.<br><br>The Phase IV regulation includes both vehicle-maximum fuel consumption limits and a corporate-average fuel consumption (CAFC) standard for each manufacturer based on vehicle curb weight distribution across the manufacturer's fleet. Manufacturers and importers must meet both standards.	2015, first introduced in 2004 and amended in 2014	Regulatory	GB 20997-2015

	China introduced fuel consumption standards for light-duty commercial vehicles in 2007 and last updated the standards in 2015. The latest standards, which are to take effect on Jan 1, 2018, are 18-27 percent more stringent than the 2007 standards and are expected to reduce the fuel consumption level of new light-duty commercial vehicles by 20 percent in 2020 as compared to 2012.<br><br>MIIT first announced its plan to develop fuel consumption standards for commercial Heavy-Duty Vehicles in 2008. The Phase 2 heavy-duty vehicle fuel consumption standards were finalized in 2014. By July 1, 2015, all new commercial HDVs sold in China (except specialized vocational vehicles) are required to comply with the Phase 2 standards.			
	**Energy-Saving and New Energy Vehicles Industry Development Plan (2012-2020)**<br>The Plan is targeting the production of 500,000 BEVs and PHEVs by 2015, with the production capacity to grow to 2 million units and the cumulative production and sales of more than 5 million of those types by 2020.<br><br>To ensure the enforcement of the Plan, the State Council issued Guidance on Accelerating the Popularization and Application of New Energy Vehicles in 2014.	2014, first released in 2012	Plan	No. 35 [2014] of the State Council
**Power**				
	**Emissions trading system (ETS)**<br>Pursuant to ***National Plan on Addressing Climate Change (2014-2020)***, a national carbon emission trading market will be formed to lower the cost of achieving GHG reduction goals.	Scheduled launch in 2017	Market	Under development
	**Implementation Plan for the Licensing System to Control Pollutant Emission**<br>The Plan requires all stationary sources of pollution in China to be licensed by 2020, to further curb emissions.	2016	Regulatory	No.81 [2016] of the State Council

	All companies should apply for the license before undertaking industrial production, allowing the authorities to monitor pollution in advance. The discharging policy gives companies a pollutant discharge permit, which covers specifics such as the variety of pollutants, concentration, and amounts allowed. Those that violate the restrictions will face stricter penalties ranging from suspension of operations to criminal charges.<br><br>The policy is scheduled to come into force by the end of 2016 in thermal power plants and papermaking companies, and then expand to cover 15 major industries which discharge air and water pollutants by 2017.			
	**Administrative Measures on Protective Full Purchase of Renewable Energy Generation**<br>The document mandates that grid companies purchase output from renewable generators, at least up to an allocated number of hours. NDRC and the NEA will be responsible for planning annual allocations of operational hours for each type of renewable generation in regions of the country that have been experiencing curtailment. Based on different circumstances, it calls on conventional power generators or the grid companies to compensate renewable energy generators for curtailment.	2016	Regulatory	NDRC Energy [2016] No. 625
	**Guiding Opinions on Promoting Electric Energy Substitution**<br>States that electric power replaces some 130 million tons of dispersed coal and fuel between 2016 and 2020, which should drive the electricity generation-to-coal consumption rate up by 1.9 percent and the electric energy-to-terminal energy consumption rate up by 1.5 percent to 27 percent.	2016	Guideline	NDRC Energy [2016] No.1054
	**Guiding Opinions on the Establishment of a Target Setting System for the Development and Utilization of Renewable Energy**<br>For the first time, portfolio standards for non-hydropower renewable energy are issued for provinces and municipalities (for the year of 2020).	2016	Regulatory	NEA [2016] No. 54<br>http://chinaenergyportal.org/en/guiding-opinions-on-the-establishment-of-a-target-setting-system-for-the-development-and-utilization-of-renewable-energy/

	**Notice on Solar PV Deployment Management and Introduction of Competitive Bidding**<br>Starting from January 1, 2016, the bidder with lowest prices (and other indicators) will be awarded the right to build a PV power plant.	2016	Fiscal	NEA New Energy [2016] No.14
	**The 13th Five Year Plan for Power Sector Development**<br>It is estimated that by 2020, Chinese electric power consumption will reach 6,800 TWh of electricity, increasing on average by 3.6-4.8% each year. The *per capita* use is expected to reach approximately 5,000 kWh by 2020. The Plan outlines specific targets for power generation from each type of energy resource as well standards for system upgrades and reforms.	2016, Last plan for the power sector was released in 2001	Plan	Document No. unknown.<br>The detailed goals can be found at:<br>http://en.cnesa.org/latest-news/2016/11/22/power-sector-reforms-announced-in-chinas-13th-five-year-Plan<br><br>Since most details of this Plan were covered in the 13th FYP for Energy Development, this document may be seen as a component of that overall guideline.
	**The 13th Five-year Plan on the Development of Renewable Energy**<br>It provides guidelines for the development of various renewable energies, including solar, wind, hydropower, biomass and geothermal energy. The Plan projects the investment for renewable energy to reach the amount of RMB2.5 trillion (approx. USD380 billion) for the 13th five-year period, and the annual usage of renewable energy will be 730 million tons of coal equivalent.<br><br>The Plan also calls for the establishment of a nationwide mechanism for trading Renewable Energy Green Certificates ("Green Certificate"), which will be used to document a power generation enterprise's use of non-hydropower renewable energy. In 2020, the electricity generated by non-hydropower renewable energy is projected to account for at least 9% for all electricity generated by each power generation enterprise.	2016; The last Mid to Long-Term Program of Renewable Energy Development and Development Plan for specific types of renewable energy were issued in 2007; Development plans for overall and specific types of renewable	Plan<br>Industrial	NDRC Energy [2016] No.2169

	In parallel to this overall plan, individual 13th Five-Year plans for hydropower, wind, bioenergy, solar, ocean energy and renewable energy development were also released.	energy were also issued for the 12th FYP in 2012.		
	**Promoting clean and efficient development of coal-fired power generation**<br>Recent policies in this regard include: ***Full Implementation of Ultra-Low Emission and Energy Saving Transformation of Coal-fired Power Plant***, ***Notice on Promoting of an Orderly Development of China's Coal-fired Power Plants (March 2016)***, ***Notice on Further Regulation of Coal-Fired Power Planning and Construction***, ***Notice on Canceling a Number of Coal Power Projects Do Not Meet Approval Conditions***, ***Notice on Further Eliminating Backward Production Capacity of Coal-fired Power Industry*** and ***Regulations of Combined Heat and Power Generation***, etc.<br><br>According to these guidelines, upgrading of coal-fired power plants to achieve ultra-low emissions and energy conservation should be completed in 2017 in the eastern region, in 2018 in the central region, and by 2020 in the western region.<br><br>A halt to construction of coal-fired plants in 13 provinces where capacity is already in surplus is ordered, including major coal producers such as Inner Mongolia, Shanxi, and Shaanxi. A further 15 provinces are required to delay construction of already-approved plants. In provinces with an electricity gap, priority should be given to the development of local non-fossil energy generation projects, with the intent to use trans-provincial energy transfers and other demand-side management approaches that could curtail the demand for new coal-fired generating plants.<br><br>Thermal power generators that have gone through many years of service and are not energy efficient, safe or environmentally sound should be phased out, and condensing units below 300 MW which have operated for at least 20 years, as well as	First policy issued in 1999, repeatedly reiterated, most recently in 2016	Regulatory Guidelines	MEP [2015] No. 164,<br>NDRC Energy [2016] No. 565,<br>NEA Power [2016] No. 244,<br>NEA Power [2016] No. 275,<br>NDRC Energy [2016] No. 855,<br>NDRC Energy [2016] No. 617

	condensation extractors for thermal power plants that have operated for 25 years or more should be shut down. Altogether, 20 GW of backward coal-fired power units are expected to be eliminated during the 13th FYP.			
	**Feed-in Tariff for Renewable Energy**<br>The latest update on FIT for renewable energy is the ***Notice on adjustments to feed-in tariffs for onshore wind and PV power*** released by the NDRC in December 2016.<br><br>According to this Notice, the 2017 benchmark feed-in tariff for PV is between 0.65 yuan/kWh ($0.094/kWh) and 0.85 yuan/kWh, depending on region – representing a cut of between 13% and 19% from 2016 levels.<br><br>The 2018 benchmark feed-in tariff for onshore wind power will range from 0.40 yuan/kWh and 0.57 yuan/kWh, representing a 15% cut from 2016 levels.<br><br>The FIT for new distributed PV is unchanged in 2017 at 0.42 RMB/kWh, as is the offshore wind rate - 0.85 RMB/kWh for offshore wind power projects and 0.75 RMB/kWh for inter-tidal wind power projects.<br><br>According to the Notice, the latest step-down in support reflects continuing reductions in deployment costs for solar and wind plants.	Updated in 2016, introduced for wind power in 2003 and updated regularly; introduced for two solar PV power plants in 2008, with rates updated regularly	Fiscal	NDRC directive [2016] No. 2729
	**Several Opinions on Further Deepening the Reform of the Electric Power System**<br>The Reform Plan seeks to encourage competition in the power sector and calls for a revamp of the existing pricing system. The Plan allows gradual infusion of social capital in the power sales and newly added distribution business, while the electricity transmission business will remain with power grid companies. Foreign capital infusion is allowed in all the fields that are not on the negative list and the same also does not need approval from the government.	2015	Guideline	No. 9 [2015] of the CPC Central Committee

	**National Solar Subsidy Program**<br>In March 2009, China announced its first solar subsidy program, the BIPV (Building-integrated photovoltaics) subsidy program, offering upfront RMB20/watt for BIPV systems and RMB15/watt for rooftop systems. In July 2009, the Golden Sun Demonstration Project, the second national solar subsidy program, was launched by the MoF, the MOST and the NEA. The project was to provide upfront subsidies for qualified demonstrative PV projects from year 2009-2012.	2009	Fiscal	MOF [2009] No.129, MOF, MOST & NEA [2009] No. 397
	**Renewable Energy Law**<br>The original law, which took effect in January 2006, was aimed at "optimizing the country's energy structure and safeguarding energy security." It covered subsidies, pricing management and supervision measures.<br><br>The revised Renewable Energy Law launches a "protective full-amount acquisition system". Although the 2005 Law contains similar requirements for state power grid enterprises to buy the total amount of power produced by renewable energy sources, it is said to be lacking in detail and therefore difficult to implement.<br><br>Electricity grid enterprises are required to reach agreements with renewable energy power-generation enterprises that have obtained administrative permits or made a filing with the government, to purchase all the renewable energy power they produce that satisfies the technical standards for grid synchronization. Power enterprises refusing to buy power produced by renewable energy generators will be fined up to an amount double that of the economic loss of the renewable energy company.	Amended in 2009, first issued in 2005	Industrial<br>Fiscal<br>Regulatory	National People's Congress, http://english.gov.cn/archive/lawsregulations/
**Industrial**				
	**Program for the Construction of an Energy-saving Standard System**<br>NDRC, Administration of Quality Supervision, Inspection and	2017	Regulatory	NDRC Environment and Resources [2017] No. 83

	Quarantine (AQSIQ) and the Standardization Administration launched ***One Hundred Energy Efficiency Standard Promotion Program*** in 2012. As of January 2017, a total of 104 compulsory energy consumption standards and 73 mandatory energy efficiency standards have been published.<br><br>This new program aims at covering all major energy intensive industries and products and enabling 80 percent of China's energy efficiency standards to be on par with international standards by 2020.			
	**The 13th Five-Year Plan for Shale Gas (2016-2020)**<br>The Plan sets the target of tapping shale gas resources trapped in reservoirs up to 3500 meters underground during these five years and producing 30 billion cubic meters of gas by 2020. The Plan also calls for further increasing production to between 80 to 100 billion cubic meters by 2030.	2016	Plan	NEA Oil & Gas [2016] No.255
	**The 13th FYP for Developing Energy Saving and Environmental Protection Industries**<br>By 2020, the added value of the energy conservation and environmental protection industries is to account for 3 percent of gross domestic product, becoming one of the pillar industries for the domestic economy.	2016, last FYP published in 2012	Guideline	No. 19 [2012] of the State Council, No. 30 [2013] of the State Council
	**Shale Gas Industrial Policy**<br>In 2016, the NEA approved ***Development Plan for Shale Gas (2016-2020)***. The NEA released China's first shale gas industrial policy in 2013, pursuant to China's ***12th Five-Year (2011- 2015) Plan for Shale Gas***. The Policy affirmed that shale gas fell within China's strategic emerging industries and called for tax incentive policies for the shale gas industry.	2016, first issued in 2013	Guideline	NEA [2013] No. 5, NEA Oil and Gas [2016] No. 255
	**Industrial Green Development**<br>In 2016, the MIIT released the ***Industrial Green Development Plan (2016-2020)*** to promote green manufacturing through green supply chain and support the fulfillment of goals set in the 13th FYP and "***Made in China 2025***".<br><br>Prior to this, ***Special Action Plan on Green Industrial Development*** was issued every year from 2012 to 2015 to	2016, first issued in 2012	Plan	MIIT [2016] No. 225

	promote the transformation and upgrade of traditional industries. Related to this, ***the 12th Five-Year Plan on Industrial Energy Conservation*** was issued in 2012.			
	**Measures for the Subsidy Funds for Energy Conservation and Emission Reduction**	2015	Fiscal	MOF [2015] No.161
	**Notice on Issuing Subsidies for Exploring and Utilizing Shale Gas**<br>In 2015, the MoF announced that a subsidy of 0.3 yuan (0.049 U.S. dollars) would be offered for every cubic meter of shale gas developed by enterprises during the 2016-2018 period, down from the 0.4 yuan provided for the 2012-2015 period. From 2019 to 2020, the subsidy will be further lowered to 0.2 yuan for every cubic meter of shale gas exploration.	Renewed in 2015, first introduced in 2012	Fiscal	MOF [2015] No.112
	**Industrial Transformation and Upgrading**<br>In 2011, the State Council disseminated the ***Plan for Industrial Transformation and Upgrading (2011-2015)*** to promote green and low-carbon industrial development.<br><br>In 2015, China sped up the optimization of the industrial structure with the introduction of "***Made in China 2025***", which sets forth such strategic tasks as improving innovative design capability, enhancing energy efficiency, promoting green transformation and upgrading and resolving overcapacity in traditional industries.	2015, previous plan issued in 2011	Plan	No. 47 [2011] of the State Council, No. 28 [2015] of the State Council
	**Action Plan for Clean and Efficient Use of Coal (2015-2020)**<br>According to the Plan, China will raise raw coal selective ratio to be above 70 percent by 2017, and 80 percent by 2020. It also Plans to cut the average coal consumption of existing coal-fired power generation units to below 310g/kwh by 2020. By then, power coal use is expected to take up more than 60 percent in total coal use. It also requires major coal-consuming sectors to improve technologies for efficient and clean use of coal and accelerate elimination of outdated furnaces and boilers.	2015	Industrial Plan	NEA [2015] No. 141
	**Regulation for Energy-Saving and Low-carbon Products Certification**	2015	Regulatory	GAQSIQ Decree No. 168

	A tentative regulation was issued by the NDRC in 2013 before the joint release of this final regulation by AQSIQ and NDRC. By March 2016, China has published two catalogues of certified products			
	**Management Measures for Certification of Energy-saving and Low-carbon Products**	Updated in 2015, first introduced in 1999	Regulatory	GAQSIQ Decree No. 168
	**Action Plan for Retrofitting and Upgrading Coal-Fired Power Plants (2014-2020)**<br>The Plan strengthens the energy efficiency and pollutants emission standards applied to coal power plants. The coal power plants with the capacity of over 600 MW are required to achieve the efficiency target of 300g of coal equivalent/kWh by 2020. The coal power plants under construction or planned are required to reach the same level of pollutants emission as natural gas power plants.	2014	Regulatory	MEP [2015] No. 164
	**Notice of Publishing the Implementation Plan for the Energy Efficiency Leader Scheme**<br>The program aims to set up a long-term mechanism to incentivize energy-efficient "leaders" and to increase the level of energy-efficiency amongst high energy-consuming products and equipment, high energy-consuming industries and public institutions. The scheme will raise current standards of energy-efficiency through incentive programs and industry benchmarks.	2014	Informative	NDRC Environment and Resources [2014] No.3001
	**Resource Tax Reform**<br>China reformed its resource tax on crude oil and natural gas in 2011, and set a new resource tax rate on coal in 2014.<br><br>Following these reforms, China began to levy a resource tax on crude oil, natural gas and coal based on the retail price rather than production to promote the more efficient use of resources. The rates were set to be between 5 to 10 percent for the crude oil and natural gas, and 2 to 10 percent for coal.	2014 and 2011	Regulatory<br>Fiscal	MOF [2011] No.114, MOF [2014] No. 72
	**Airborne Pollution Prevention and Control Action Plan (2013-2017)**	2013	Regulatory	No. 37 [2013] of the State Council

	The Plan proposes to improve overall air quality across the nation through five years, reduce heavy pollution by a large margin and make obvious improvement of air quality in Beijing-Tianjin-Hebei Province, the Yangtze River Delta and the Pearl River Delta. By 2017, the level of inhalable particulate matter in cities above prefecture level is to be dropped by at least 10% against 2012 level and the days with good air quality will be increased year on year. The level of fine particulate matter in Beijing-Tianjin-Hebei Province, the Yangtze River Delta and the Pearl River Delta will be cut by 25%, 20% and 15% respectively and the annual concentration of fine particulate matter in Beijing will be kept at 60 mcg /m^3.<br><br>Coal consumption as a proportion of the entire energy mix should decline from the current 68 percent to 65 percent in 2017.			
	**Guideline Catalogue for Industrial Restructuring**<br>To achieve China's target for conserving energy and reducing emissions by optimizing and upgrading its industrial structure.	Updated in 2013, first issued in 2005	Informative	NDRC directive [2013] No. 21
	**The Capacity Elimination Program**<br>The level of capacity cuts for most of the industries rose substantially in 2010, along with the issuance of the ***Notice of the State Council on Further Strengthening the Elimination of Backward Production***. In 2012, the MIIT issued the ***Notice of Issuing the Objectives of Eliminating Backward Production Capacity in 19 Industrial Sectors***. The five industries with severe excess capacity identified in 2012 include steel, cement, aluminum, flat glass, and shipbuilding. In 2013 and 2014, the first and second lists were issued of enterprises to be eliminated in the 19 industrial sectors. In 2013, the State Council issued the ***Guidelines to Tackle Serious Production Overcapacity***, laying out the measures to deal with the problem.<br><br>During the 13FYP, China plans to phase out 800 million tons of backward coal capacity per year while adding 0.5 billion tons per year advanced production capacity. Meeting its 2016	2013, national level order first issued in 1999 and became a staple of government proclamations in 2007, with specific lists of affected factories and categories being updated regularly	Regulatory	No. 7 [2010] of the State Council, MIIT directive [2012] No. 159, No. 41 [2013] of the State Council

	target ahead of schedule, China plans to reduce steel output by an additional 100 million tons to 150 million tons by 2020.			
	**Promotion of Energy Efficient Products and Technologies** Since 2012, MIIT has published every year an ***Energy Efficiency Star Certified Product Catalogue***. Specific energy efficient product or technology catalogues have been issued for electromechanical devices, electronic motors, industrial boilers, internal-combustion engine, energy-saving technologies in telecommunication and other low-carbon technologies.	2012	Informative Voluntary	MIIT [2016] No.59
	**Top-1000 (then Top-10,000) Enterprises Program** China's mandatory energy conservation target-setting policy for large energy users, known as the Top-10,000 Program was introduced in 2011 under the 12th FYP, as an expansion of its successful predecessor, the Top 1,000 Program which operated between 2006 and 2010. The Top-10,000 Program covered two-thirds of China's total energy consumption. It achieved the energy-saving target of 250 million tons of coal equivalent (Mtce) one year before the 2015 deadline.	2011, first introduced in 2006	Regulatory Voluntary	NDRC Environment and Resources [2006] No. 571 NDRC Environment and Resources [2011] 2873
	**Energy-Saving Products Benefiting the Public** The project provides a promotion list of energy-efficient products which covered three major categories including household appliances, vehicles and industrial products, fifteen varieties and about 100,000 types of energy-efficient products. The central government allocated more than 40 billion yuan for the implementation of the project.	2009, concluded in 2013	Fiscal Regulatory	MOF & NDRC [2009] No.213
	**Differential Electricity Price Policy** The policy requires a punitive electricity price (a surcharge of 0.2 yuan/kwh) on eight energy intensive industries. In 2010, the NDRC further increased punitive electricity prices on energy intensive industrial enterprises. The electricity consumed by outdated enterprises will be charged a surcharge of 0.1 yuan/kwh and electricity consumed by restricted enterprises will be charged an additional 0.3 yuan/kwh.	2006	Regulatory	No.77 [2006] of the General Office of State Council
**Green Finance**				
	**Guiding Opinions on Building a Green Financial System**	2016	Guideline	No. 228 [2016] of the People's Bank of China

	The Guidelines include a series of policy incentives to support and incentivize green investment. These incentives include re-lending operations by the People’s Bank of China, specialized green guarantee programs, interest subsides for green loan-supported projects, the support for introduction of the PPP model in the green industry, and the launch of a national-level green development fund.<br><br>The Guidelines emphasizes the importance for building and improving the unified rules and regulations for green bonds, and taking measures to reduce the financing cost of green bonds.<br><br>The Guidelines also emphasizes the development of Green Insurance and requires a further expansion in international cooperation on Green Finance.			
	**Preferential Tax Policies for Renewable Energy**<br>In 2001 value added tax (VAT) for wind power cut in half, to 8.5% (normal rate 17%). In the same year, a circular determined that VAT collected for using municipal solid waste for power generation would be refunded back to the producer. In 2003, the VAT for biogas production was also reduced to 13%.<br><br>Since 2005, the VAT levied on small-scale hydropower plants has been 6% of the income and the VAT collected from some large hydropower plants have been returned to the companies. Fuel ethanol produced by certified enterprises has been exempted from consumption tax and VAT.<br><br>The import duties and import VAT have been gradually exempted for renewable energy equipment imported by domestic and later on foreign invested renewable energy projects.<br><br>Since the enactment of China’s new Corporate Income Tax in 2008, tax breaks have been granted to companies with	2016, first introduced in 2001, with new policies being added over the years	Fiscal	MOF & SAT [2016] No.81

	synergistic utilization of resources or investment in technology for environmental protection, energy and water conservation.<br><br>In 2013, MoF announced 50 percent of the VAT refund to producers of solar PV power. The policy was extended in 2016. Newly-added wind farms after 2015 are eligible for a 50 percent refund on their VAT.			
	**Energy Efficiency Credit Guidelines**<br>The Guidelines put forward feasible instructions in terms of the characteristics of energy efficiency projects, the priorities in energy efficiency credit businesses, business entry, key issues in risk examination, process management, product innovation, etc.	2015	Guideline	CBRC & NDRC [2015] No.2
	**Green Financial Bond Directive**<br>The directive as well as the associated Green Bond-Endorsed Project Catalogue set out the official requirements for what projects qualify as green, management of proceeds and reporting.	2015	Guideline	No. 39 [2015] of the People's Bank of China
	**Interim Measures on the Management of the Additional Renewable Energy Surcharge Fund**<br>They set the rates of subsidies for the operation and maintenance costs to be incurred by connecting renewable energy to the grid – depending on the distance of power transmission: 0.01 RMB/kWh for less 50 km, 0.02 RMB/kWh for 50-100 km, and 0.03 RMB/kWh for 100 km or longer.	2012	Fiscal	MOF [2012] No. 102
	**Green Credit Guidelines**<br>The Guidelines encourage Chinese banks to lend more to energy efficient and environmentally sustainable companies and less to polluting and high energy consuming enterprises. The Guidelines require banks to measure and control environmental and social risks in lending and will be applied to all lending – both domestic and overseas.	2012	Guideline	CBRC [2012] No.4
	**Interim Measures for the Administration of the Collection and Use of the Renewable Energy Development Fund**<br>The renewable energy development fund shall include the special-purpose fund appropriated by the public budget of the national finance and the income from surcharges on renewable	2011	Fiscal	MOF [2011] No. 115

	energy power prices as collected from power users according to law.			
	**Provisional Measures for Administration of the Financial Reward Capital for the Innovation of Energy-saving Technology**<br>The prerequisite for getting the rewards is that companies must have consumed more than 20,000 tonnes of coal equivalent (TCE) per year before adopting energy-efficient technologies, and save 5,000 tonnes of coal equivalent (TCE) after their tech-transformation. Companies in eastern regions will be rewarded with 240 yuan ($36.92) per TCE saved while those in central and western regions will be given 300 yuan per TCE saved.	Revised in 2011, first issued in 2007	Fiscal	MOF & NDRC [2011] No.367
**Land use**				
	**Plan on National Forest Management (2016-2050)**<br>The Plan sets the objective of raising the forest cover to over 26 percent by 2050 an increasing the forest stock volume to 23 billion cubic meters.	2016	Plan	SFA [2016] No.88
	**The 13th Five Year Plan for China's National Forestry Development (2016-2020)**<br>The Plan envisages China's national forest stock will reach 1.4 billion cubic meters, the forest cover will rise to just over 23 percent and the total value of forestry industry output will amount to RMB8.7 trillion.	2016	Plan	SFA [2016] No.22
	**Instruction Opinions on Advancing the Forestry Carbon Sink Trade**	2014	Guideline	SFA [2014] No.55
	**Outlines of National Forestation Plan (2011-2020)**<br>The Plan sets the targets of increasing forest coverage to over 23 percent, forest stock volume to over 15 billion cubic meters, forestry output to 10 trillion RMB and compulsory tree planting rate to 70 percent by 2020.	2011	Plan	NAC & SFA [2011] No.6
	**The Program of Constructing a National Monitoring System on Forest Sinks**<br>The SFA issued the ***Interim Management Measures for Quantifying and Monitoring Forest Carbon Sinks*** in 2010 and started to build the forest inventory system in 17 pilot provinces/municipalities.	2010	Informative	SFA [2010] No.26

	By the end of 2015, the program had covered 25 provinces, autonomous regions and municipalities, Xinjiang Production and Construction Corps, as well as the country's four biggest forest industry groups. A basic database of forestry carbon sinks was built up.			
	**China's Forestry Action Plan to Deal with Climate Change** This Action Plan stipulates the goals of three stages. By 2050, the forest area will realize a net growth of 47 million hectares compared with 2020, and the carbon-sequestering ability of forests of the country will reach a stable level. This Action Plan stipulates 22 major actions of the forestry industry, including 15 actions for mitigating climate change and 7 actions for adapting to it.	2009	Plan	Serial number unknown
**Residential and Commercial**				
	**The 13th Five-year Plan for the Development of Energy Efficient and Green Building** The objectives are to improve the energy efficiency for all new urban residential and public buildings by 20 percent compared with 2015 while building energy conservation standards in some regions and for key architectural components such as windows and doors shall meet or come very close to the current international advanced level by 2020. Green buildings shall account for more than 50 percent of all new urban buildings with the use of energy-saving construction materials exceeding 40 percent. Energy efficiency renovation need to be carried out for more than 500 million m^2 of existing residential buildings and 100 million m^2 of public buildings. Among existing residential buildings in cities and towns across the country, at least 60 percent shall achieve green building standards.	2017, plan for the previous five-year plan period issued in 2012	Plan	MOHURD [2017] No.53
	**The Solar Energy for Poverty Alleviation Programme** The programme aims to add over 10 GW capacity and benefit more than 2 million households from around 35,000 villages across the country by 2020.	Updated in 2016, first introduced in 2014	Fiscal	NDRC Energy [2016] No.621
	**Design Standards and Acceptance Codes for Residential and Public Buildings**	Updated in 2015, first	Regulatory	GB50189-2015

	China's first energy efficient building design standard was implemented in 1986 for buildings in the northern heating zones, with the standard being updated in 1995 and again in 2010. China also issued the Code for Acceptance of Energy Efficient Building Construction in 2007. The Acceptance Code makes compliance with building energy efficiency requirements mandatory for the final acceptance of a construction project.	introduced in 1986		
	**Green Building Evaluation Standard (The Three Star System)** The system grants three levels, with 3-star being the highest rated green buildings. The updated green building standard provides evaluation protocols to differentiate between residential buildings and public-purpose buildings, and to provide bonus points for ongoing improvements.	Revised in 2014, first introduced in 2006	Informative Voluntary	GB - T50378-2014
	**Standard for Lighting Design in Buildings** For some building types, the maximum lighting power density values defined by the new Chinese standard are slightly lower than values defined by the Building Area Method in ASHRAE 90.1-2013 of the United States.	Updated in 2013, first introduced in 2004	Regulatory	GB 50034-2013
	**Green Building Action Plan** The Plan puts forward the goal of completing 1 billion sq. m. of new green buildings during the period of the 12th Five Year Plan and demanding the full implementation of green building standards from 2014 for government-invested buildings, public housing in major cities and large public-purpose buildings with an area of more than 20,000 sq. m.	2013	Plan	No.1 [2013] of the General Office of the State Council
	**Implementation Guidance on Accelerating the Development of Green Buildings in China** The Guidance specifies that 2-Star green buildings enjoy a subsidy of RMB 45 per sq. m. and 3-Star green buildings enjoy a subsidy of RMB 80 per sq. m (construction area).	2012	Fiscal	MOF & MOHURD [2012] No.167
	**Roadmap for the Phasing Out of Incandescent Bulbs** The Roadmap pledged to replace the 1 billion it uses annually with more energy efficient models within five years. As of October 1, 2016, incandescent bulbs above the 15-watt range	2011	Regulatory	NDRC [2011] No.28

	were required to be eliminated from all retailers, completing the five-year task.			
	**Energy Conservations in Public Institutions**<br>In 2008 the State Council issued the ***Regulation on Energy Conservation by Public Institutions***, a key regulation describing the responsibilities and requirements of all public institutions for improving energy efficiency.<br><br>The ***12th FYP for Energy Conservation in Public Institutions*** published in 2011 includes the targets of reducing public institutions' energy consumption per person by 15 percent and the unit energy consumption for building floor area by 12 percent by 2015 as compared to 2010.	2011 and previously in 2008	Regulatory	NGOA Energy Conservation [2011] No.433
	**Circular on Establishing System of Compulsory Government Procurement of Energy Conservation Products**<br>Up to June 2017, NDRC and MoF have published 22 editions of the government procurement lists.	2007	Regulatory	No.51 [2007] of the General Office of the State Council
**Provincial or Local Policies**				
	**Mandatory Coal Cap Targets**<br>The Airborne Pollution Prevention and Control Action Plan unveiled by the State Council in 2013 pledged to cap coal consumption and improve the air quality of the entire country by 2017. Thereafter, six Chinese provinces have established absolute coal consumption reduction targets in their air pollution action plans, with a 50% reduction targeted in Beijing, 13% in Hebei, 19% in Tianjin, 5% in Shandong, 21% in Chongqing and 13% in Shaanxi, by end of 2017, compared to 2012 levels.	Various dates Between 2013-2017	Regulatory	No.37 [2013] the General Office of State Council
	**Low Carbon City**<br>In 2010 the Low Carbon City Pilot Scheme was formally endorsed by the NDRC The main aims of the scheme was to develop low-carbon dioxide emission industries, establish a greenhouse gas emission data collection and management system, and encourage residents to adopt green consumption patterns. In 2012, a total of 6 provinces and 36 cities were involved in the scheme. NDRC issued another 45 low carbon	Updated in 2017, first introduced in 2010	Guideline	NDRC Climate [2017] No.66

	pilot cities at the beginning of 2017, thus raising the total number of low carbon provinces and cities to 87.			
	**Carbon Emissions Trading Pilot Programs**<br>In 2011, the NDRC approved to establish carbon emissions trading pilot programs in seven provinces or cities (Beijing, Chongqing, Shanghai, Tianjin, Guangdong, Hubei, and Shenzhen).<br><br>Following Shenzhen launching its trading in June 2013, Shanghai, Beijing, Guangdong, and Tianjin, in turn, launched their first trading before the end of 2013. The remaining two of the seven pilot schemes, Hubei and Chongqing, launched trading on 2 April and 19 June 2014, respectively.	2011	Market	NDRC [2011] No. 2601
